# Influenza B-cells Protective Epitope Characterization: A Passkey for the Rational Design of New Broad-Range Anti-Influenza Vaccines

**DOI:** 10.3390/v4113090

**Published:** 2012-11-14

**Authors:** Nicola Clementi, Elena Criscuolo, Matteo Castelli, Nicasio Mancini, Massimo Clementi, Roberto Burioni

**Affiliations:** Microbiology and Virology Unit, “Vita-Salute” San Raffaele University, Milan 20132, Italy; Email: criscuolo.elena@hsr.it (E.C.); m.castelli@studenti.unisr.it (M.C.); mancini.nicasio@hsr.it (N.M.); clementi.massimo@hsr.it (M.C.); burioni.roberto@hsr.it (R.B.)

**Keywords:** monoclonal antibody, protective epitopes, heterosubtipic neutralizing activity, epitope-based influenza vaccine.

## Abstract

The emergence of new influenza strains causing pandemics represents a serious threat to human health. From 1918, four influenza pandemics occurred, caused by H1N1, H2N2 and H3N2 subtypes. Moreover, in 1997 a novel influenza avian strain belonging to the H5N1 subtype infected humans. Nowadays, even if its transmission is still circumscribed to avian species, the capability of the virus to infect humans directly from avian reservoirs can result in fatalities. Moreover, the risk that this or novel avian strains could adapt to inter-human transmission, the development of resistance to anti-viral drugs and the lack of an effective prevention are all incumbent problems for the world population. In this scenario, the identification of broadly neutralizing monoclonal antibodies (mAbs) directed against conserved regions shared among influenza isolates has raised hopes for the development of monoclonal antibody-based immunotherapy and “universal” anti-influenza vaccines.

## 1. Introduction

The outbreak of the highly pathogenic avian influenza (HPAI) H5N1 isolates highlighted how much influenza viruses are still a serious threat for human health. Four influenza A pandemics occurred since 1918 (H1N1 in 1918, H2N2 in 1958, H3N2 in 1967 and again H1N1 in 2009), whereas several other subtypes (H5N1, H9N2, H7N7) have caused concern for the possible broad transmission to humans. Human infections by H5N1 avian viruses were first reported in China [1,2,3,4]. Since then, this subtype has spread among poultry and wild birds from Asia to Europe and Africa [5]. So far, 608 human cases, resulting from direct contact with poultry and birds, and 359 deaths have been reported by the WHO (World Health Organization) (Source: WHO/GIP, data in HQ as of 10 August 2012). 

The genetic reassortment events (antigenic shift) occurring periodically represent a concern, as recently shown by the 2009 H1N1 pandemic [6,7,8]. Moreover, the continuous generation of viral antigenic variants (antigenic drift) is not only the major cause of the seasonal epidemics, but also the factor determining the emergence of isolates resistant to currently available anti-influenza drugs (adamantanes and neuraminidase inhibitors) [9,10,11,12]. Given this background, the rapid availability of “universal” prophylactic or therapeutic tools is of extreme importance [5,7,13,14,15,16].

To overcome the limitations of the current vaccinal approaches, different strategies for the development of novel vaccinal strategies have been proposed [5,7,17,18,19,20,21,22]. A pivotal role in the rational design of novel broadly protective approaches can be played by the fine definition of B-cell epitopes on influenza hemagglutinin (HA), widely shared among phylogenetically highly divergent influenza subtypes. This can be achieved by using broadly neutralizing monoclonal antibodies (mAbs) as “molecular probes”. In this review, we describe the epitopes of a panel of mAbs endowed with heterosubtypic neutralizing activity and able to target conformational motifs widely shared among influenza isolates.

## 2. Hemagglutinin and Protective mAbs

Influenza HA is an homotrimeric envelope protein, constituting two subunits (HA1 and HA2), featuring two different functions during viral replication: it binds the cellular receptor by its globular head and, after the endocytosis-mediated entry, it allows the fusion between the viral envelope and the endocytic vesicle membrane by its stem region.

The monoclonal antibodies (mAbs) directed against the HA globular head, formed by the HA1 subunit, have been crucial to understand its antigenic organization. In particular, using mouse mAbs and structural analysis, five distinct antigenic sites have been identified on H3N2 HA [23,24,25], on H1N1 HA [26] and on H2N2 HA [27]. However, it was recently reported that the structures of antigenic sites of H5N1 HA [28,29] and H9N2 HA [30] may be different from those described for H1, H2, and H3 subtypes [31]. These sites are very prone to mutate due to the high selective pressure exerted by the humoral immune response. For these reasons, Abs directed against these highly variable antigenic sites usually feature only homologous (or, less frequently, homosubtypic) neutralizing activity, that is, with few exceptions [32,33], directed against a very limited panel of closely related viruses.

The highly hydrophobic fusion stem region of HA (mainly formed by HA2) is less prone to mutate. An high conservation rate has been observed among all influenza subtypes, belonging to phylogenetic group 1 (H5, H1, H2, H9, H6, H8, H11, H12, H13 and H16) and group 2 (H3, H7, H4, H10, H14 and H15) [34]. This region is less exposed to the immune system, and therefore less immunogenic than the immunodominant HA globular head. This implies that the antibody response directed against this HA portion, if present, only represents a minority compared to the whole anti-HA humoral response. Moreover, the proximity of this region to the cellular membrane can lead to a negative selection of potentially auto-reactive B cell clones [35,36,37]. Nevertheless, the availability of mAbs directed against the HA stem has allowed to demonstrate that this region is very important for the protection against viral isolates belonging to different influenza subtypes (heterosubtypic neutralizing activity). Indeed, among the panel of mAbs directed against the different influenza virus proteins [38,39,40,41,42,43,44,45,46,47,48,49,50], the most promising both for clinical use and epitope-based vaccine design are those directed against the stem [51,52,53,54,55,56,57,58,59,60,61].

## 3. Broadly Neutralizing mAbs: Dual Role in the Fight Against a Variable Virus

Table 1 reports the main biological features of the most promising heterosubtypic mAbs described to date. Most of the mAbs originate from a single VH-gene subfamily (VH1-69) often associated with autoimmune diseases [62,63,64,65]; the very broadly neutralizing mAbs FI6v3 and PN-SIA28 belong to VH3-30 [53,54], whereas PN-SIA49 to VH3-23 [55]. All these mAbs feature unique biological activity and can certainly be considered for a possible future use in clinical prophylactic or therapeutic practice [65,66,67,68,69,70,71,72,73], for laboratory diagnosis [74,75,76,77,78] or, regarding the topic of this review, as a “probe” for the identification of B-cell protective epitopes for novel vaccine design approaches [79,80,81,82,83].

**Table 1:** Panel of mAbs endowed with heterosubtypic neutralizing activity. “✘” indicates the neutralization activity assessed against the isolates belonging to the different subtypes. The IC_50_ (half maximal (50%) inhibitory concentration) indicates the concentration of mAb required for 50% inhibition *in vitro*. mAbs able to recognize highly divergent influenza subtypes are highlighted by black box. Red box indicates that all the mAbs are able to recognize epitopes on H5N1 HA.

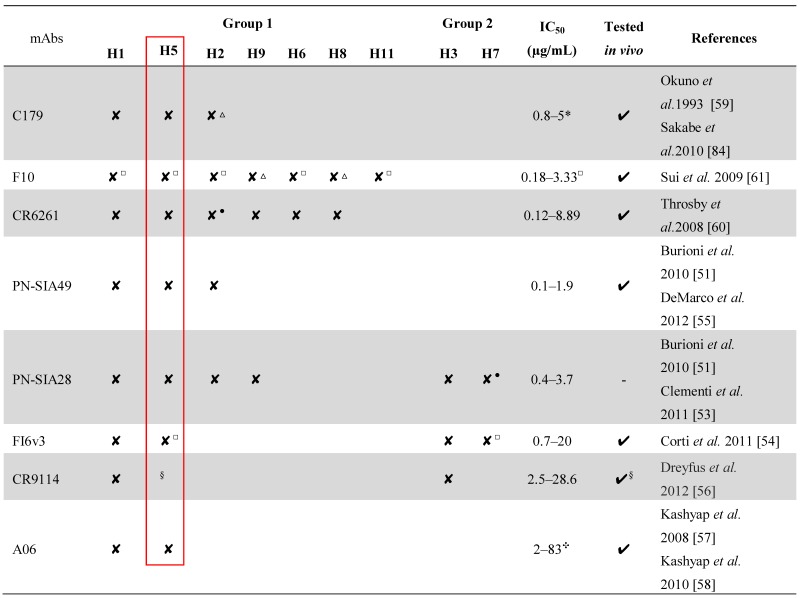

^*^: Neutralizing activity index: was determined by dividing the virus titers (log_10_TCID_50_/mL) in the presence of PBS by those in the presence of C179.^△^: IC_50_ not calculated. ^●^: IC_50 _> 10 µg/mL. ^□^: Neutralization activity calculated using pseudovirus-based assays. ^§^: Binding assays and co-crystal structure generation on H5. *In vivo* protection against influenza B. ^✣^: Minimum Inhibitory Concentration (MIC).

### 3.1. “Classical” Vaccine Limitations

The “classical” vaccinal strategies are based on the use of killed or attenuated microorganisms, or of their purified antigens (Ags) [85]. Unfortunately, these vaccinal approaches present several drawbacks when coping with the hypervariability of influenza viruses [5]. The choice of the influenza isolates to be included in the new vaccine preparations (seasonal vaccine) is made by analyzing the sequences of previously circulating influenza strains and evaluating their antigenic profile. Moreover, the time for vaccine production is strictly related to the time needed for culturing the chosen strain on embryonated eggs, requiring several months to reach the amount needed. Finally, the emergence of completely new isolates cannot be predicted, as demonstrated by the 2009 pandemic which highlighted the limits of the current vaccine manufacturing technologies [5]. Similarly, the emergence of a potentially pandemic HPAI isolate could not be easily faced with the classical vaccine production strategy [5].

The trend in the development of novel strategies is mainly focused on the setting up of vaccine preparations containing only the “universally” protective epitopes, through the fine definition of the B-cell epitopes recognized on HA by unique heterosuptypic neutralizing mAbs. The identification of the three dimensional conformational motifs constituting these epitopes could lead to the generation of small molecules [86,87,88] that can actually mimic them (mimotopes) and elicit a broadly protective Ab response *in vivo*.

## 4. B-Cell Epitopes Widely Shared among Different HA Subtypes

The fine definition of a protective epitope is crucial for the effectiveness of a new vaccine preparation.

The most promising mAbs useful to reach this result are those endowed with broad range neutralizing activity (Table 1). Their epitopes have been widely characterized using different approaches such as peptide panning, alanine scanning, *in vitro* generation of viral escape mutants under the selective pressure of the mAb of interest, competitions between mAbs for the binding to HA, binding assays and co-crystal structure generation [30,52,53,54,55,56,59,60,61]. Below, we provide three different analyses of the HA regions bound by these mAbs, performed in order to visualize, describe and compare, under different point of views, the epitopes recognized by them. Finally, a sequence analysis of the residues involved in the above epitopes on H5N1 isolates is reported.

### 4.1. Epitope Mapping

**Figure 1:**
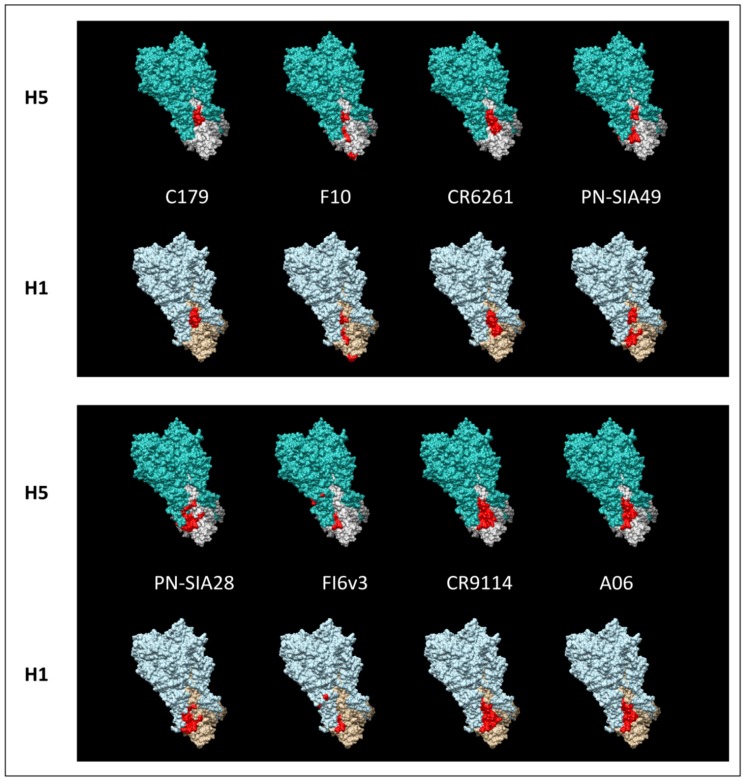
Mapping of the different B-cell epitopes (red) on the crystal structures of trimeric HAs belonging to H5 and H1 subtypes (pdb id number 2FK0 and 1RU7). HA1 and HA2 are depicted respectively in light green and white for H5 subtype and light blue and beige for H1 subtype.

**Figure 2:**
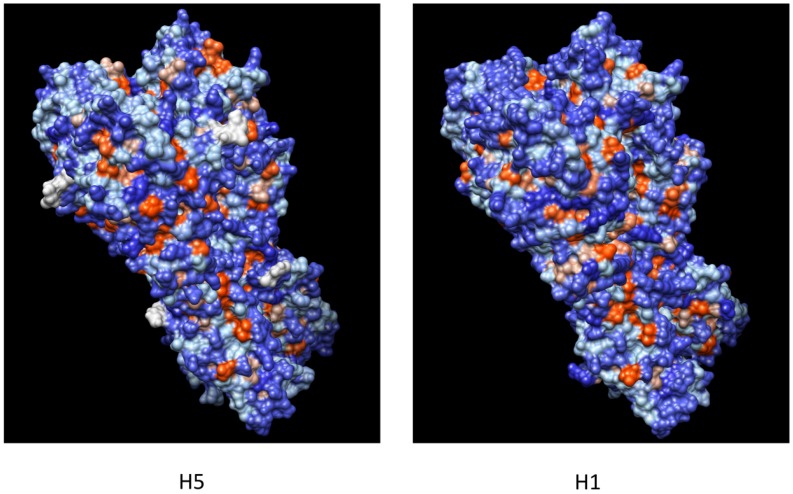
Crystal structures of influenza HAs (H5 and H1). The color transition (red to blue) indicates the different hydrophobic (red) and hydrophilic (blue) regions present on the HAs. Analysis performed using the Kyte-Dolittle scale.

The mapping of the different epitopes on the crystal structures of HAs belonging to H5 and H1 subtypes (A/Viet Nam/1203/2004 and A/Puerto Rico/8/1934), highlighted in Figure 1, shows that all the broadly neutralizing mAbs recognize epitopes on the HA stem. All the epitopes encompass overlapping residues belonging to HA2, and in most cases to the HA1 subunit as well (Figure 1). The spatial conformation of the epitopes on HA is similar in both subtypes. These epitopes are characterized by a buried hydrophobic fusion peptide surrounded by mainly hydrophilic solvent-exposed surrounding areas (Figure 2). The location of the epitopes well correlates with the inhibition of the fusion activity of HA, that is, the neutralizing mechanisms suggested for each mAb. 

### 4.2. Epitope Conservation among Subtypes

**Figure 3:**
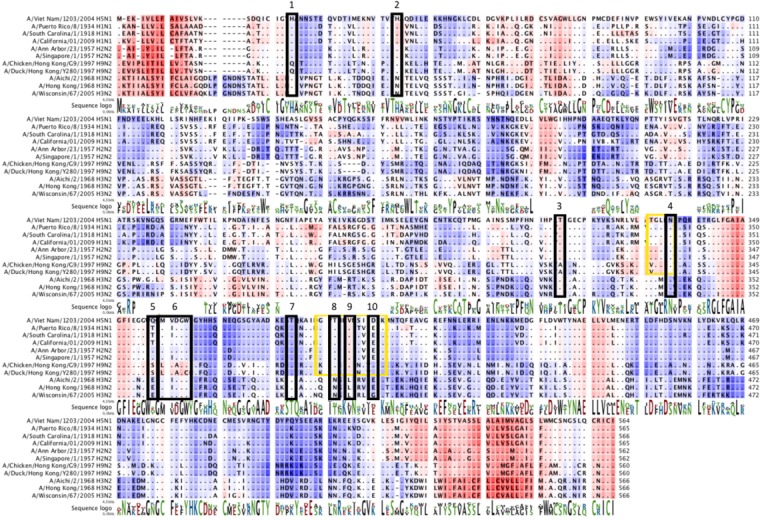
Multiple sequence alignment: sequence logo shows amino acid conservation. The sequence hydrophobicity profile is indicated by gradient color (red most hydrophobic) in background, black and yellow boxes underline two example of conserved epitopes belonging respectively to PN-SIA28 (neutralizing both group1 and 2) and C179 (only group 1).

Aligning the HA sequences belonging to the different influenza subtypes, it is possible to evidence two amino acid conservation patterns among group 1 and group 2 viruses (sequence logo in Figure 3). These conservation patterns partially justify the different biological activity of the mAbs that can be divided into two groups: the mAbs solely directed against group 1 viruses (C179, F10, CR6261, PN-SIA49 and A06) [51,52,55,57,58,59,60,61] and those directed against both group 1 and 2 (PN-SIA28, FI6v3 and CR9114) [51,52,53,54,56]. As an example, the epitopes recognized by C179 and PN-SIA28 are highlighted by yellow and black boxes, respectively, in Figure 3. Regarding PN-SIA28 epitope, it is possible to identify residues shared among all the HAs (group 1 and 2) involved in its binding (Figure 3, boxes 4 and 7 in black). Interestingly, differences within the PN-SIA 28 epitope between the two HA groups (black box 2 in Figure 3), have been shown to reduce, but not to abrogate, PN-SIA 28 binding to group 2 HA [53]. This example suggests that amino acid differences in a single position does not necessarily disprove the importance of that residue for HA cross-recognition, suggesting that a mere HA sequence study (performed without considering experimental observations regarding the different mAb biological activities) may not evidence HA regions able to elicit a cross-subtype protection.

**Figure 4:**
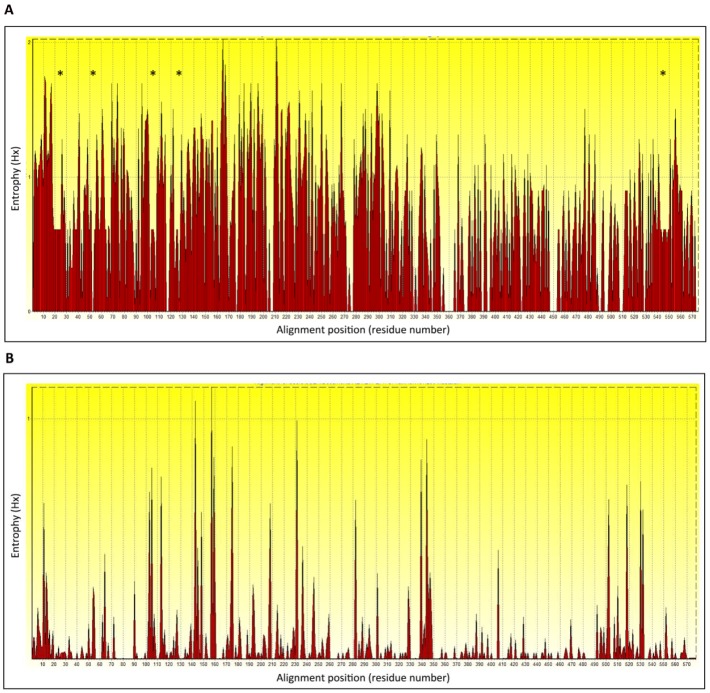
Entropy plot calculated for a large number of influenza HA amino acid sequences.(**A**) Analysis performed on H1, H2, H5, H9 and H3 subtypes. Asterisks indicate several sequence regions differing between influenza phylogenetic group 1 and 2. (**B**) Entropy plot obtained for HA belonging to H5N1 subtype.

On the other hand, a sequence study can certainly represent a simple starter point for the selection of HA regions in which amino acid residues constituting the protective epitopes are highly shared among all isolates, as epitope-based vaccine backbone. Moreover, an entropy plot of the different HA sequences can give an idea of the amount of variability through a definite sequence position in an alignment. More accurately, it measures the lack of predictability for an alignment position and gives a measure of uncertainty at each position relative to other positions [89]. The entropy plot calculated for a large number of HA amino acid sequences belonging to the different influenza subtypes (Figure 4A) highlights the matching of several amino acid residues belonging to the epitopes above described with the most conserved residues in the HA sequences of the different influenza isolates (group 1 and group 2).The HA regions differing between group 1 and 2 are highlighted by truncated peaks (asterisks in Figure 4A). In particular, one of these regions (second asterisk, Figure 4A) encompasses part of PN-SIA28 epitope and includes the amino acid highlighted by black box 2 in Figure 3. The same analysis performed using up to 300 H5N1 isolates (human and avian) demonstrates the presence of HA regions less prone to mutate also on the H5N1 isolates (Figure 4B), suggesting that the HA regions recognized by these mAbs can elicit a protective humoral response directed against a plurality of H5N1 isolates.

## 5. H5N1 Ccross-Clade Protection

**Figure 5:**
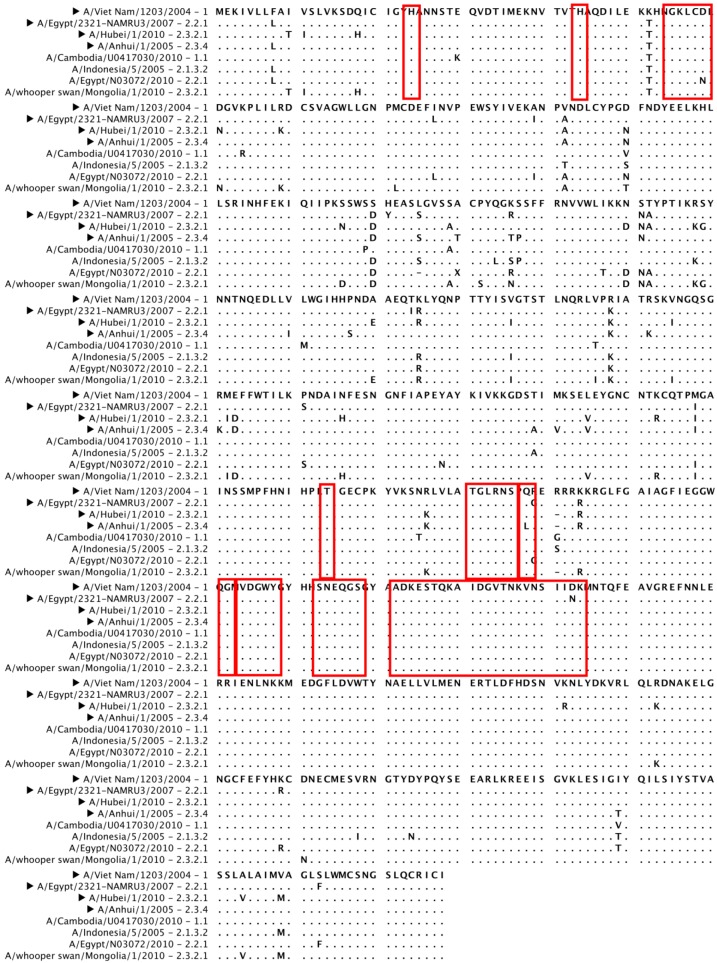
Amino acid sequence alignment of H5N1 HAs. The HA of several candidate vaccine viruses (►) belonging to different H5N1 clades have been aligned to HAs of isolates belonging to several H5 clades currently circulating [90,91]. Red boxes indicate the epitopes of the broadly-neutralizing mAbs described in this review able to neutralize H5N1 viruses.

Avian H5N1 viruses continue to spread among animals and, more limitedly, humans, continuing to evolve and diversify. H5N1 isolates are phylogenetically divided into clades (0 to 9) and subclades on the basis of their HA sequence [90]. The currently circulating H5N1 isolates that have infected humans can be grouped in four different clades (1.1, 2.1.3.2, 2.2.1 and 2.3.2.1) (WHO report February 2012) [91]. The divergence between different clades and subclades can correlate with a different antigenic pattern allowing the viral escape from the selective pressure of neutralizing antibodies directed against another antigenic group [19]. Several H5N1 isolates, belonging to different clades, have been identified as putative strains to be included in a possible pre-pandemic vaccine [91]. Aligning some of them with several currently circulating H5N1 isolates responsible of human infections (Figure 5) it is possible to observe that there are homology regions shared among the isolates. These regions are coherent with the HA portions less prone to mutate identified by the entropy plot calculation performed for H5N1 sequences (Figure 4B); more interestingly, several homology regions (most of them solvent exposed on the 3-D structure of H5-HA) encompass the epitopes recognized by the broadly neutralizing mAbs described in this review (red boxes in Figure 5; H5-HA in Figure 2). This underlines the possible cross-clade protective potential of the HA regions recognized by the mAbs if used to develop a new class of molecules to be included in new vaccine preparations able to confer a cross-clade protection.

## 6. Conclusions

Influenza pandemics represent a severe problem for the world’s population. The major natural reservoirs for the influenza virus reassortment are animals. Nowadays, particular attention is reserved for the avian reservoir, which cannot be efficiently controlled (wild birds), and at the same time represent an economical problem in case of infections (poultry). In both cases, even considering a potential transmission route involving an intermediate host, the emergence of viral strains able to infect humans and potentially able to cause pandemics is a chief menace for the human health. An example of a lethal case of avian-human transmission route is the H5N1 outbreaks, which threaten the public health. Moreover, the health and economic balance burdened by the influenza seasonal epidemics constitutes an additional issue for humans. In fact, both epidemics and pandemics can only be definitely defeated by an universal vaccine. Unfortunately, to date, the approaches adopted to reach this main goal have not been conclusive. An essential contribution could be the rational design of anti influenza vaccines through epitope-based strategies. In this field, the use of mAbs endowed with broad neutralizing activity as a tool for a deeper knowledge of the regions able to elicit a similar protective immune response could be the key to success.
